# Ribosome biogenesis-related gene signature predicts prognosis and immune landscape in glioma and identifies UTP20 as a therapeutic target

**DOI:** 10.3389/fimmu.2025.1680667

**Published:** 2025-10-08

**Authors:** Yadan Li, Xiaolong Tang, Wenhui Zhao, Xiuwen Si, Yanfang Cui, Yanbin Dong, Yongshuo Liu

**Affiliations:** ^1^ Department of Clinical Laboratory, Binzhou Medical University Hospital, Binzhou, Shandong, China; ^2^ Department of Laboratory Medicine, Hospital of Chengdu University of Traditional Chinese Medicine, Chengdu, Sichuan, China; ^3^ Department of Clinical Laboratory, The Affiliated Lianyungang Municipal Oriental Hospital of Kangda College of Nanjing Medical University, Lianyungang, China; ^4^ Department of Pathology, The Affiliated Lianyungang Hospital of Xuzhou Medical University, The First People’s Hospital of Lianyungang, Lianyungang, China; ^5^ Department of Clinical Laboratory, Shandong Cancer Hospital and Institute, Shandong First Medical University and Shandong Academy of Medical Sciences, Jinan, Shandong, China

**Keywords:** glioma, ribosome biogenesis, UTP20, prognostic outcome, therapeutic susceptibility

## Abstract

**Background:**

Glioma, the most prevalent primary brain tumor, exhibits dysregulated ribosome biogenesis closely linked to malignant behavior. However, the role of ribosome biogenesis in glioma and prognosis remains incompletely understood. This study aimed to construct a molecular signature based on ribosome biogenesis-related genes to predict patient survival and therapeutic response in glioma.

**Methods:**

Utilizing The Cancer Genome Atlas (TCGA) glioma cohort data, we constructed a ribosome biogenesis-related genes (RBRGs) signature using LASSO regression and multivariate Cox analyses, and subsequently validating its prognostic value in independent cohorts. We systematically evaluated the signature’s associations with clinicopathological features, tumor immunity, genomic instability, tumor stemness, and therapeutic sensitivity. The oncogenic role of the key gene UTP20 was experimentally validated in U87 and U251 glioma cell lines through MTS, colony formation, and transwell assays.

**Results:**

We established a four-gene RBRGs signature (NOP10, UTP20, SHQ1, and PIH1D2). Elevated RBRGs score significantly correlated with shortened overall survival and adverse clinical characteristics, including advanced age, high WHO grade, IDH wild-type status, and absence of 1p/19q codeletion. A nomogram incorporating the RBRGs score demonstrated excellent predictive performance (C-index = 0.841). RBRGs-associated genes were enriched in immune regulatory pathways. The high-risk group exhibited increased infiltration of immunosuppressive cells (macrophages, myeloid-derived suppressor cells [MDSCs], and cancer-associated fibroblasts [CAFs]), upregulation of immunosuppressive checkpoints, and resistance to immunotherapy. Furthermore, the RBRGs signature correlated with genomic alterations, heterogeneity, tumor stemness, and therapeutic sensitivity. Crucially, UTP20 knockdown significantly suppressed glioma cell proliferation and invasion *in vitro*.

**Conclusion:**

The RBRGs signature was successfully developed and validated as an independent prognostic biomarker and predictor of therapeutic response in glioma, highlighting its extensive association with tumor heterogeneity. Furthermore, this study identified UTP20 as a key oncogenic driver that facilitates glioma progression.

## Introduction

Gliomas, the most common primary malignant tumors of the central nervous system, originate from neuroglial precursor cells and can differentiate into multiple subtypes, including astrocytomas, oligodendrogliomas, ependymomas, and oligoastrocytomas (1). According to the molecular-histopathological stratification defined by the WHO 2021 classification framework for central nervous system tumors (CNS5), gliomas are categorized into low-grade (WHO grades I–II) and high-grade (III–IV) types. Among them, glioblastoma (GBM, grade IV) is the most aggressive and lethal phenotype, characterized by its ability to infiltrate brain tissue by crossing the blood-brain barrier, and it demonstrates significant resistance to treatment (2). Paradoxically, despite revolutionary advancements in contemporary multimodal neuro-oncological approaches, including surgical resection, targeted molecular therapies, and radiation and chemotherapy regimens, the five-year overall survival rate for patients with high-grade gliomas remains exceedingly low (with the five-year overall survival rate for glioblastoma patients being less than 10%) (3, 4). This underscores the urgent need for the identification of novel molecular biomarkers and the establishment of molecularly stratified prognostic frameworks to overcome current therapeutic limitations.

The emergence of multi-omics technologies, particularly single-cell transcriptomics and spatial genomics, has been widely applied in cancer treatment prediction, facilitating not only enhanced survival prediction accuracy via machine learning-optimized algorithms but also the identification of glioma-specific therapeutic targets (5, 6). Importantly, these models have become indispensable for quantifying dynamic tumor-immune interactions and predicting responses to immune checkpoint blockade, thereby accelerating the transition from empirical therapies to molecularly stratified treatment strategies in neuro-oncology.

Ribosome biogenesis, an evolutionarily conserved cellular program coordinating ribosomal RNA transcription, post-transcriptional processing, and ribosomal subunit assembly, serves as a master regulator of cellular proliferative homeostasis. Its dysregulation is now recognized as a hallmark of neoplastic transformation (7). Tumor cells exploit this machinery via pathological hyperactivation, as exemplified in glioblastoma by RPS6-driven enhancement of cancer stemness programs that promote invasive progression and therapeutic resistance (8). Mechanistically, m6A-related RNA modifiers and serine/arginine-rich splicing factors cooperatively boost ribosome biogenesis, establishing feedforward pathways that perpetuate treatment resistance in glioma subtypes (9–11). Despite these critical insights into gliomagenesis, systematic investigation of ribosome biogenesis-related genes (RBRGs) as clinically actionable prognostic biomarkers in glioma remains largely unexplored.

Leveraging the comprehensive multi-omics datasets from The Cancer Genome Atlas (TCGA) consortium, this investigation established a robust prognostic signature centered on ribosome biogenesis-related genes, identifying four pivotal biomarkers (NOP10, UTP20, SHQ1, and PIH1D2). These genes exhibited significant transcriptional upregulation in glioma tissues compared to normal brain parenchyma. Patients classified into the high-risk group showed markedly poorer clinical outcomes, underscoring the prognostic significance of dysregulated ribosome biogenesis in glioma pathobiology. Subsequent integrative analyses revealed strong associations between the RBRGs score and distinct features of the tumor immune microenvironment (TIME), particularly the infiltration patterns of tumor-associated macrophages (TAMs), myeloid-derived suppressor cells (MDSCs), and cancer-associated fibroblasts (CAFs), highlighting its potential clinical utility in predicting sensitivity to immunotherapy. Furthermore, substantial divergences in genomic alteration landscapes and pharmacological sensitivity profiles were identified between the defined RBRGs subgroups, providing a novel theoretical framework for advancing personalized precision medicine approaches in glioma management.

## Materials and methods

### Data source

Transcriptomic profiles and corresponding clinical prognostic records for glioma patients were curated from comprehensive public repositories, including The Cancer Genome Atlas (TCGA) (https://portal.gdc.cancer.gov/), the Chinese Glioma Genome Atlas (CGGA) (http://www.cgga.org.cn/), and the Gene Expression Omnibus (GEO) (https://www.ncbi.nlm.nih.gov/geo/) (**Table 1**). A cohort of 331 ribosome biogenesis-related genes, identified based on a prior publication (12) was subsequently analyzed for their association with tumor characteristics (**Supplementary Table S1**).

**Table 1:** Details of the cohorts used in this study.

Database	Function	Total
TCGA	Training set	tumor (n=765), normal (n=5)
CGGA301	Prognostic validation set	tumor (n=301)
CGGA325	Prognostic validation set	tumor (n=325)
GSE43378	Prognostic validation set	tumor (n=50)
GSE16011	Expression validation set	tumor (n=276), normal (n=8)
PRJNA482620	Prognostic validation set	tumor (n=34)
GSE91061	Prognostic validation set	tumor (n=109)

### Derivation of a ribosome biogenesis-related genes signature

Utilizing the TCGA glioma cohort, a prognostic signature based on ribosome biogenesis-related genes was constructed through an integrated analytical framework. Initial survival screening via the R package “survival” identified prognosis-associated genes, followed by least Absolute Shrinkage and Selection Operator regression using R package “glmnet” applied to RBRG-overlapping candidates to mitigate overfitting. Subsequent univariate and multivariate Cox regression analyses, conducted using the R “survival” package, refined the model parameters, establishing a multivariate-derived RBRGs signature. The chromosomal positions of the four core RBRGs (NOP10, UTP20, SHQ1, and PIH1D2) were visualized using R package “RCircos”, and inter-gene correlations were assessed through *Spearman* analysis implemented via R package “circlize”. Patients were classified into RBRGs-high and -low groups based on the median RBRGs score. Risk stratification and predictive accuracy were evaluated using risk factor scatter plots generated with R package “ggplot2”, Kaplan-Meier survival analysis performed with R packages “survminer” and “survival”, and time-dependent receiver operating characteristic (ROC) curves constructed using R packages “timeROC” and “ggplot2”.

Validation of the RBRGs signature was systematically conducted across independent cohorts. Differential expression analysis of the four core RBRGs (NOP10, UTP20, SHQ1, and PIH1D2) and RBRGs score in glioma versus normal brain tissue was first confirmed in TCGA and GSE16011 transcriptomic datasets. The prognostic robustness of the multivariate-derived RBRGs was subsequently assessed in three independent glioma cohorts (CGGA301, CGGA325, and GSE43378) through three-tiered analytical validation: (1) risk factor scatter plots, (2) Kaplan-Meier survival estimation with log-rank testing, and (3) time-dependent ROC curve analysis quantifying predictive accuracy at multiple time points, with all methods implemented as previously described.

### Clinical relevance of the RBRGs signature and construction of nomogram

The clinical relevance of the RBRGs signature was investigated through stratified analysis of RBRGs distributions across key clinicopathological variables, including gender, age, WHO grade, IDH mutational status, and 1p/19q codeletion status, within the TCGA and CGGA693 glioma cohorts. A prognostic nomogram was established by integrating multiple variables, including the RBRGs score, age, WHO grade, IDH mutation status, and 1p/19q codeletion status, using the TCGA cohort via the SangerBox platform (http://vip.sangerbox.com/), thereby enabling individualized survival prediction in glioma patients. Nomogram calibration was evaluated by comparing observed and predicted survival probabilities, while dynamic predictive accuracy over time was assessed using time-dependent ROC analysis implemented via R packages “timeROC” and “ggplot2”.

### Functional enrichment analysis

Functional annotation of the RBRGs signature was performed through integrated transcriptomic profiling of TCGA glioma cohorts, stratified by the median RBRGs score, while retaining genes with an expression level ≥10 counts in at least 10% of the samples. Differential gene expression analysis between RBRGs-high and -low groups was conducted using the R package “DESeq2”, generating false discovery rate (FDR)-adjusted significant differentially expressed genes (DEGs). The RBRGs-associated differentially expressed genes (DEGs) were subjected to Gene Ontology (GO) enrichment analysis using the R package “clusterProfiler”, with gene identifier conversion performed via R package “org.Hs.eg.db”. Functional relevance was quantified through Z-score calculations using R package “GOplot”, and the results were visualized as multi-panel bar and circle plots generated with R package “ggplot2”. Complementary pathway-level insights were derived via gene set enrichment analysis (GSEA) using hallmark gene sets curated from the molecular signatures database (MSigDB) Collections (https://www.gsea-msigdb.org/gsea/msigdb/); enrichment statistics computed by R package “clusterProfiler” were rendered as publication-quality plots through R package “ggplot2”.

### Tumor microenvironment analysis

Comprehensive characterization of the glioma immune microenvironment was performed through multi-algorithmic deconvolution of TCGA transcriptomes. Immune cells infiltration landscapes were quantified using the CIBERSORTx (Cell-type Identification By Estimating Relative Subsets Of RNA Transcripts, extended version) (https://cibersortx.stanford.edu/) platform with LM22 reference matrix, revealing differential abundances of 22 leukocyte subsets between RBRGs-stratified groups. Concurrently, correlations between the four core RBRGs (NOP10, UTP20, SHQ1, and PIH1D2) and immune fractions were established. The tumor immune dysfunction and exclusion (TIDE) framework further delineated tumor immune evasion features through comparative assessment of myeloid-derived suppressor cells (MDSCs) and cancer-associated fibroblasts (CAFs) distributions. Stromal and immune compartmentalization was objectively measured via ESTIMATE scores, followed by correlation analysis with the four core RBRGs. Predefined immune subtypes underwent Kaplan-Meier survival validation to confirm prognostic stratification utility (13). Single-cell resolution of the four core RBRGs expression was achieved by interrogating the GSE131928 cohort through tumor immune single-cell hub 2 (TISCH2) (http://tisch.comp-genomics.org/), mapping cell type-specific transcriptional patterns across the glioma ecosystem.

### Immunotherapy predictive capability analysis

Predictive utility of the RBRGs signature for immunotherapy response was systematically evaluated through multi-platform interrogation of glioma immunity networks. The seven stages of the cancer-immunity cycle, comprising antigen release, dendritic cell presentation, T cell priming and activation, immune trafficking, tumor infiltration, cancer cell recognition, and cytotoxic killing, were profiled using the tracking tumor immunophenotype (TIP) database (http://biocc.hrbmu.edu.cn/TIP/) to quantify immune dysregulation associated with RBRGs. *Spearman* correlation analysis leveraging TCGA transcriptomes established significant associations between four core RBRGs (NOP10, UTP20, SHQ1, and PIH1D2) and clinically actionable immune checkpoint molecules. Multi-dimensional stratification integrating RBRGs thresholds with expression levels of key checkpoint regulators (CTLA4, PDCD1, CD274, HAVCR2, PDCD1LG2, TNFRSF4, and TNFRSF18) revealed distinct survival outcomes validated by Kaplan-Meier analysis. External validation using TIDE algorithm, GSE91061 and PRJNA482620 cohorts further confirmed the RBRGs signature’s capacity to discriminate immunotherapy resistance.

### Genetic mutation, genomic heterogeneity, and tumor stemness analyses

Multi-dimensional genomic profiling of TCGA glioma encompassed mutation spectrum characterization, tumor heterogeneity quantification, and stemness evaluation. Somatic mutation landscapes were analyzed using the R package “maftools”, which identified the top 15 significantly mutated genes within each RBRGs subgroup based on integrated variant data retrieved from the GDC portal (https://portal.gdc.cancer.gov/). Kaplan-Meier analysis compared overall survival disparities between mutant- versus wild-type carriers of key drivers (EGFR, PTEN, NF1, IDH1, CIC, and ATRX), with parallel assessment of the four core RBRGs expression (NOP10, UTP20, SHQ1, and PIH1D2) shifts across genotypes. Tumor mutation burden (TMB) was quantified from non-synonymous variants using the R package “maftools”, while microsatellite instability (MSI) indices were calculated based on established published criteria (14). Genomic instability metrics, including tumor purity, ploidy, homologous recombination deficiency (HRD) scores, and neoantigen load (13), were correlated with RBRGs score. Finally, five established stemness indices (RNAss, EREG-METHss, DMPss, ENHss, and EREG-EXPss) (15) were investigated for their association with the RBRGs score, revealing mechanistic links between ribosome biogenesis and malignant cellular plasticity.

### Chemotherapy and radiotherapy sensitivity analyses

Therapeutic sensitivity profiling combined computational prediction of chemosensitivity with correlation analysis of radiotherapy response. For conventional chemotherapy, dose-response landscapes of first-line glioma agents were modeled using the core algorithm of R package “oncoPredict”, which mechanistically integrates drug pharmacokinetics with TCGA transcriptomic signatures. This analysis revealed statistically significant differential sensitivities between RBRGs subgroups. Regarding radiotherapy, Response evaluation criteria in solid tumors (RECIST)-categorized treatment outcomes were leveraged to compare the RBRGs score distributions among non-responders (progressive/stable disease) versus responders (partial/complete response) within irradiated TCGA cohorts, with SangerBox-derived visualizations quantifying the RBRGs signature’s association with therapeutic resistance.

### Cell culture and siRNA transfection

Glioma cell lines U87, U251 were cultured in Dulbecco’s modified Eagle’s medium (DMEM, Gibco) supplemented with 10% fetal bovine serum (FBS, Gibco) and 1% penicillin-streptomycin at 37 °C in 5% CO2. siRNAs were purchased from Sangon Biotech. The sequences of the siRNAs were provided in **Supplementary Table S2**. Glioma cells were seeded in 12-well plates and transfection was initiated when the cell density reached approximately 70%. Transfection was performed using Lipofectamine 2000 (Invitrogen, 1668019) according to the manufacturer’s instructions.

### RNA isolation and qPCR

Total RNA was extracted using Trizol reagent (Invitrogen, A33251), and reverse transcription was conducted using M-MLV Reverse Transcriptase (Promega, M1701). Quantitative real-time PCR was carried out in triplicate using the SYBR Green Master Mix (Yeasen, 11203ES08). The relative gene expression was assessed by normalizing the expression of each target gene to GAPDH and calculated using the 2^(-△△Ct) method. The following primers were provided in **Supplementary Table S2**.

### Western blot analysis

Western blot analysis was performed as previously described (16). Briefly, glioma cells were lysed using 1× SDS sample buffer (25 mM Tris-HCl, pH 6.8, 1% SDS, 5% Glycerol) supplemented with protease inhibitor cocktail. Equal amounts of protein were separated by 8% SDS-PAGE and transferred to PVDF membrane (Millipore, IPVH00010). The membranes were blocked with 5% skimmed milk and then incubated overnight at 4 °C with primary antibodies: UTP20 (Proteintech, 18830-1-AP) and β-actin (Abmart, P30002). Following primary antibody incubation, the membranes were incubated with the secondary antibody at room temperature for 1 hour. Protein bands were visualized using the Super ECL detection reagent (Pierce, 32106).

### Cell proliferation, colony formation and invasion assays

Cell proliferation was evaluated using the MTS assay (Promega, G3580), in accordance with the manufacturer’s instructions. Absorbance at 490 nm was measured at 0, 24, 48, 72, and 96 h to determine cell viability. For the colony formation assay, 1×10³ cells were plated in 6-well plates and cultured for 14 days. Following the incubation, the cells were fixed with 4% paraformaldehyde and stained with 0.1% crystal violet to visualize the colonies. In the invasion assay, 5×10^4^ cells were placed into Transwell inserts with an 8 μm pore size, pre-coated with Matrigel. After 24 hours of incubation, the invasive cells were stained with 0.1% crystal violet, examined under a microscope, and the number of invading cells was quantified using ImageJ software.

### Statistical analysis

All statistical analyses were processed on R Studio (v4.3.3) or GraphPad Prism (v10.1.2) platforms, and P value < 0.05 indicated statistically significant differences. The quantitative results were presented as the mean ± standard deviation (SD). *Wilcoxon rank sum* test was used for unpaired samples, *t*-test was used for paired samples, and *ANOVA* was used for comparisons between multiple groups. *Log-rank* test was used for Kaplan-Meier survival analysis. *Spearman* test was used for Correlation analysis.

## Results

### Construction of a ribosome biogenesis-related gene signature in glioma

**Figure 1:**
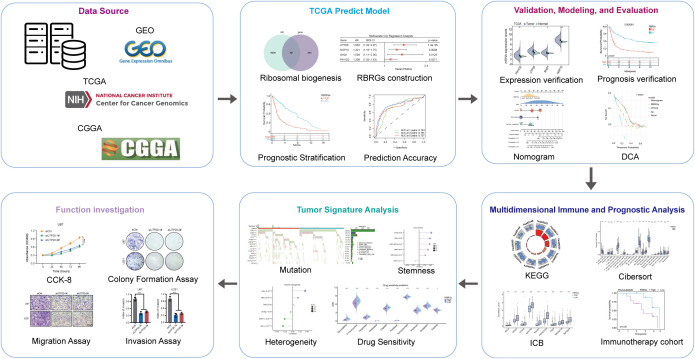
Flow chart of this study.

**Figure 2:**
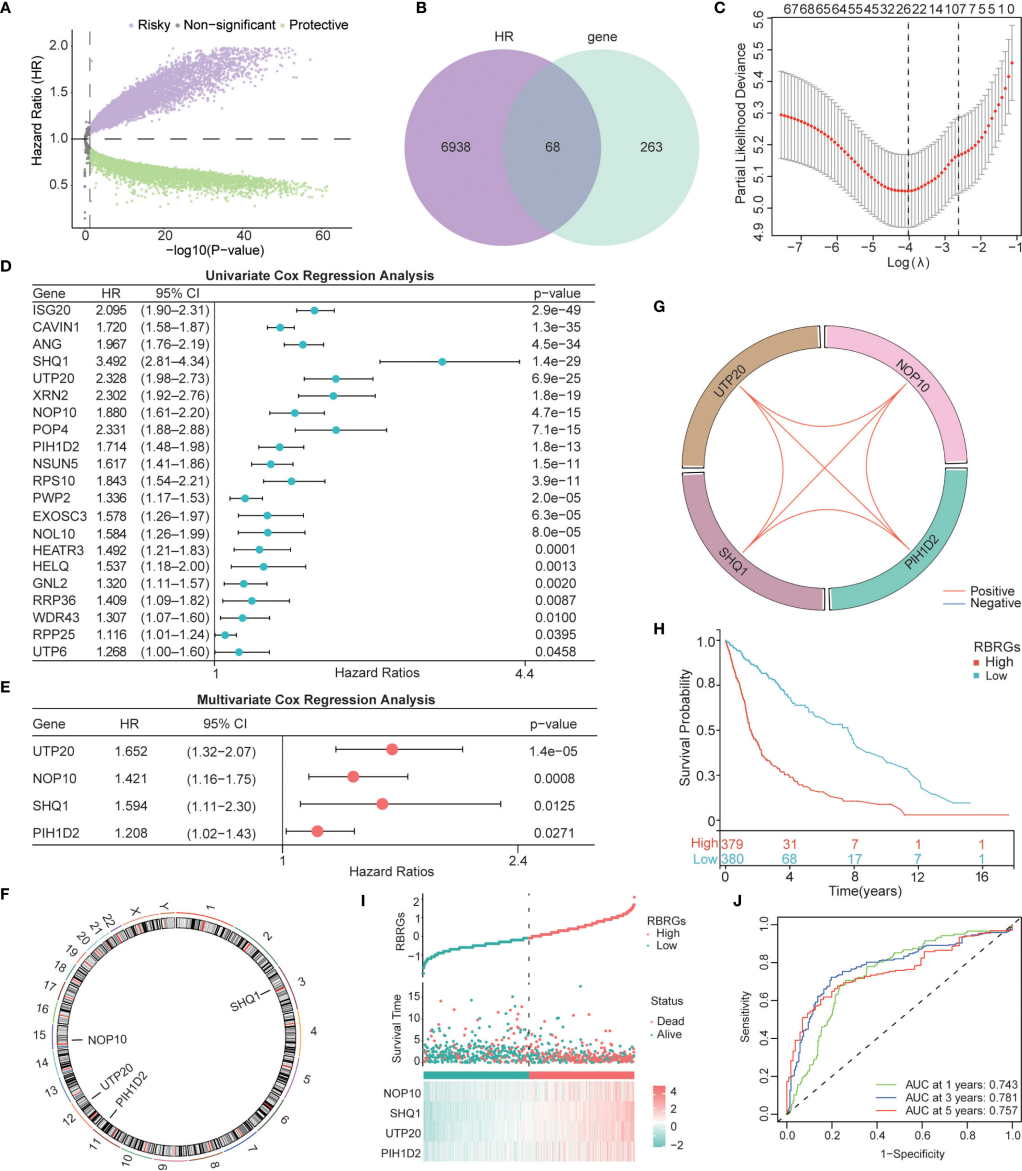
Construction of a Ribosome biogenesis-related gene signature based on the TCGA glioma cohort. **(A)** Volcano map displayed the genes that affected the survival of glioma patients. **(B)** Venn diagram showing the intersection of 331 RBRGs with risky genes from TCGA. **(C)** LASSO regression analysis narrowing down to 22 candidate genes. **(D)** Univariate and **(E)** multivariate Cox regression selecting 4 independent prognostic genes. **(F)** Genomic map showing specific localization of NOP10, UTP20, SHQ1, and PIH1D2 in chromosomes. **(G)** Circos plot showing the correlation among these 4 genes. **(H)** Kaplan–Meier survival analysis of glioma patients in the RBRGs-high and -low groups using the TCGA cohort. **(I)** Scatter plot demonstrating survival time and number of deaths in the two groups. **(J)** Time-dependent ROC curves demonstrating the predictive accuracy of RBRGs for 1-, 3-, and 5-year survival in glioma patients.

The workflow of this study was depicted in **Figure 1**. To develop a prognostic signature based on ribosome biogenesis-related genes in glioma, we utilized the TCGA glioma cohort as the training dataset. Initial analysis identified 7,006 genes associated with increased risk and 8,247 genes associated with decreased risk (**Figure 2A**). Intersection of the 7,006 high-risk genes with 331 known RBRGs yielded 68 overlapping candidates (**Figure 2B**). Subsequent application of LASSO regression refined this set to 22 potential prognostic genes (**Figure 2C**). Univariate and multivariate Cox proportional hazards regression analyses identified four core RBRGs (NOP10, UTP20, SHQ1, and PIH1D2) as independent prognostic biomarkers (**Figures 2D, E**). These genes were incorporated into a risk score model defined by the formula: RBRGs score = (0.35 × NOP10 expression) + (0.50 × UTP20 expression) + (0.47 × SHQ1 expression) + (0.19 × PIH1D2 expression). Genomic Map indicated the chromosomal locations of these genes as follows: NOP10 at 15q14, UTP20 at 12q24.31, SHQ1 at 3q26.3, and PIH1D2 at 17p13.1 (**Figure 2F**). Circos plot analysis revealed significant positive correlations among the expression levels of these four genes within glioma tissues (**Figure 2G**).

Patients stratified into RBRGs-high and -low groups based on the median RBRGs score exhibited significantly divergent clinical outcomes. Kaplan-Meier survival analysis demonstrated markedly inferior overall survival (OS) for the RBRGs-high group compared to the -low group (**Figure 2H**). A scatter plot further illustrated that higher RBRGs score correlated strongly with increased mortality and shorter survival times (**Figure 2I**). The predictive power of the RBRGs signature was robustly validated using time-dependent ROC analysis, yielding AUC values of 0.743, 0.781, and 0.757 for 1-, 3-, and 5-year OS, respectively (**Figure 2J**). These results confirm the strong prognostic capacity of this RBRGs signature in glioma.

### Validation of the RBRGs signature in glioma

**Figure 3:**
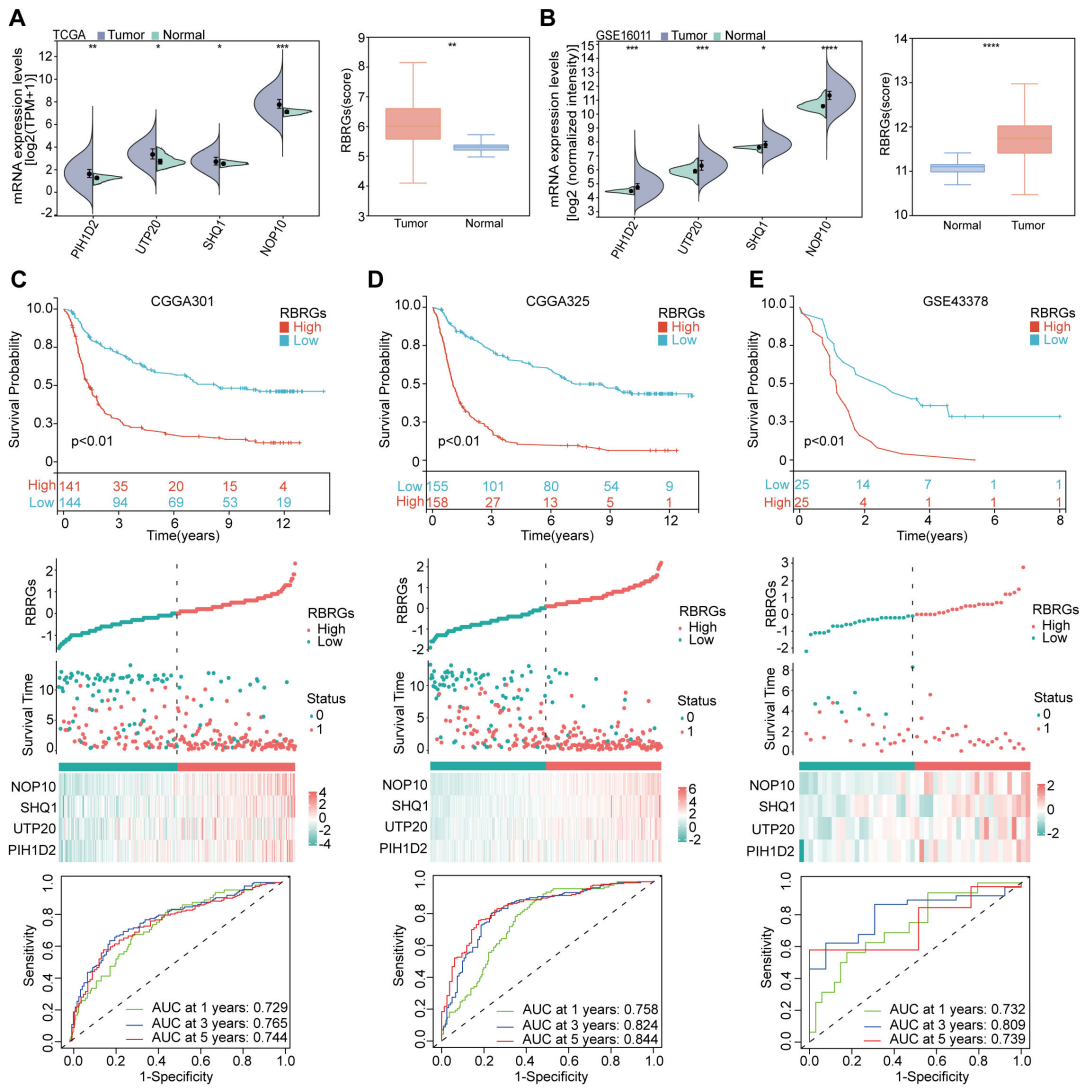
Validation of RBRGs expression levels and prognostic value was based on multiple cohorts. **(A, B)** TCGA and GSE16011 cohorts were used to validate the expression levels of NOP10, UTP20, SHQ1, PIH1D2, and RBRGs in normal and glioma tissues, respectively. **(C–E)** The Kaplan-Meier curves, scatter plots, and time-dependent ROC curves were utilized to validate the prognostic value of RBRGs in glioma using the CGGA301, CGGA325, and GSE43378 cohorts. *p < 0.05, **p < 0.01, ***p < 0.001, ****p < 0.0001.

We first validated the expression patterns of the signature components (NOP10, UTP20, SHQ1, and PIH1D2) and the integrated RBRGs score in glioma versus normal tissues. Analysis of TCGA and GSE16011 cohorts demonstrated significant upregulation in tumor tissues compared to normal controls (**Figures 3A, B**). To assess the prognostic robustness, we evaluated the RBRGs signature across three independent glioma cohorts (CGGA301, CGGA325, and GSE43378). Consistent with the training data, patients with high-risk score exhibited significantly shorter overall survival than those with low scores in all validation sets, accompanied by substantially elevated mortality rates. Time-dependent ROC analysis further confirmed the RBRGs signature’s predictive power, with AUC values exceeding 0.700 for 3- and 5-year survival predictions across all cohorts (**Figures 3C–E**). Collectively, these results demonstrated the signature’s consistent prognostic accuracy across diverse patient populations.

### Optimization of the glioma prognostic model

**Figure 4:**
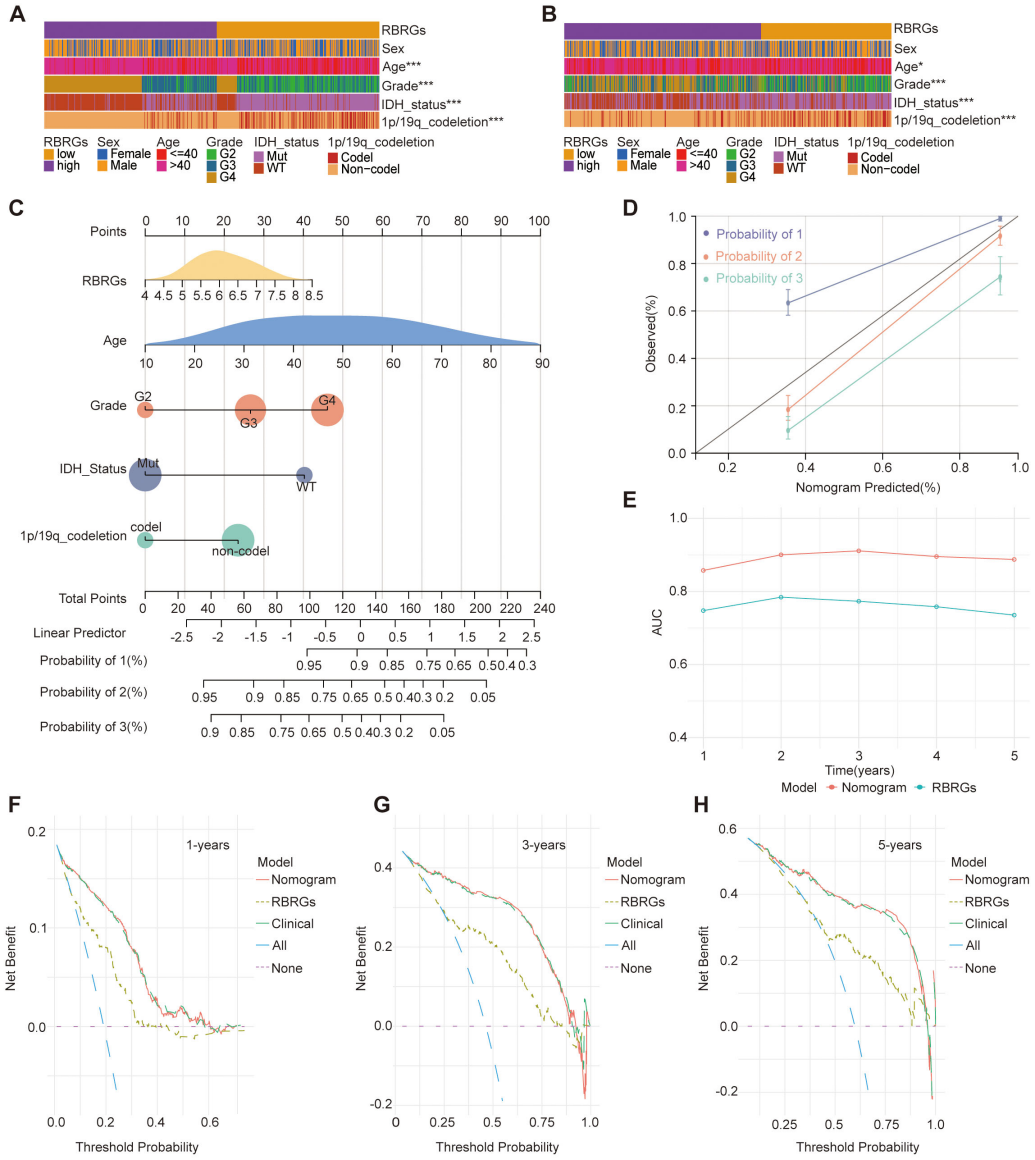
Clinical relevance of RBRGs and optimization of the model. **(A, B)** Differences in clinicopathologic characteristics of glioma patients between the RBRGs-high and -low groups utilized the TCGA and CGGA databases, including sex, age, WHO grade, IDH status, and 1p/19q codeletion. **(C)** Five variables, RBRGs, age, WHO grade, IDH status, and 1p/19q codeletion, were used to construct the Nomogram model based on the TCGA glioma cohort. **(D)** Calibration curves. **(E)** Time-dependent AUC curves. **(F–H)** DCA curves for 1-, 3-and 5-year, respectively. *p < 0.05, ***p < 0.001.

To enhance the predictive accuracy of our RBRGs signature, we investigated key clinicopathological factors influencing the prognosis of glioma patients. Comparative analysis of TCGA and CGGA693 cohorts revealed significant associations between the RBRGs score and critical prognostic variables, including patient age, WHO grade, IDH mutation status, and 1p/19q codeletion, while no significant correlation was observed with gender (**Figures 4A, B**). Integrating these covariates with the RBRGs score, we constructed a comprehensive nomogram for individualized survival prediction (**Figure 4C**). The model demonstrated high discriminative power, with a concordance index (C-index) of 0.841 (95% CI: 0.820-0.862), supported by calibration curve (**Figure 4D**). Time-dependent ROC analysis further validated its robustness, showing AUC values > 0.700 for 1- to 5-year overall survival predictions (**Figure 4E**). Clinically relevant decision curve analysis (DCA) revealed superior net benefit of the nomogram compared to the RBRGs signature alone and negative/positive QC lines, particularly at 3- and 5-year time points (**Figures 4F–H**). Collectively, the integration of clinicopathological variables with the molecular signature significantly enhances prognostic precision and clinical utility in glioma management.

### Functional crosstalk between the RBRGs signature and immune signaling pathways

**Figure 5:**
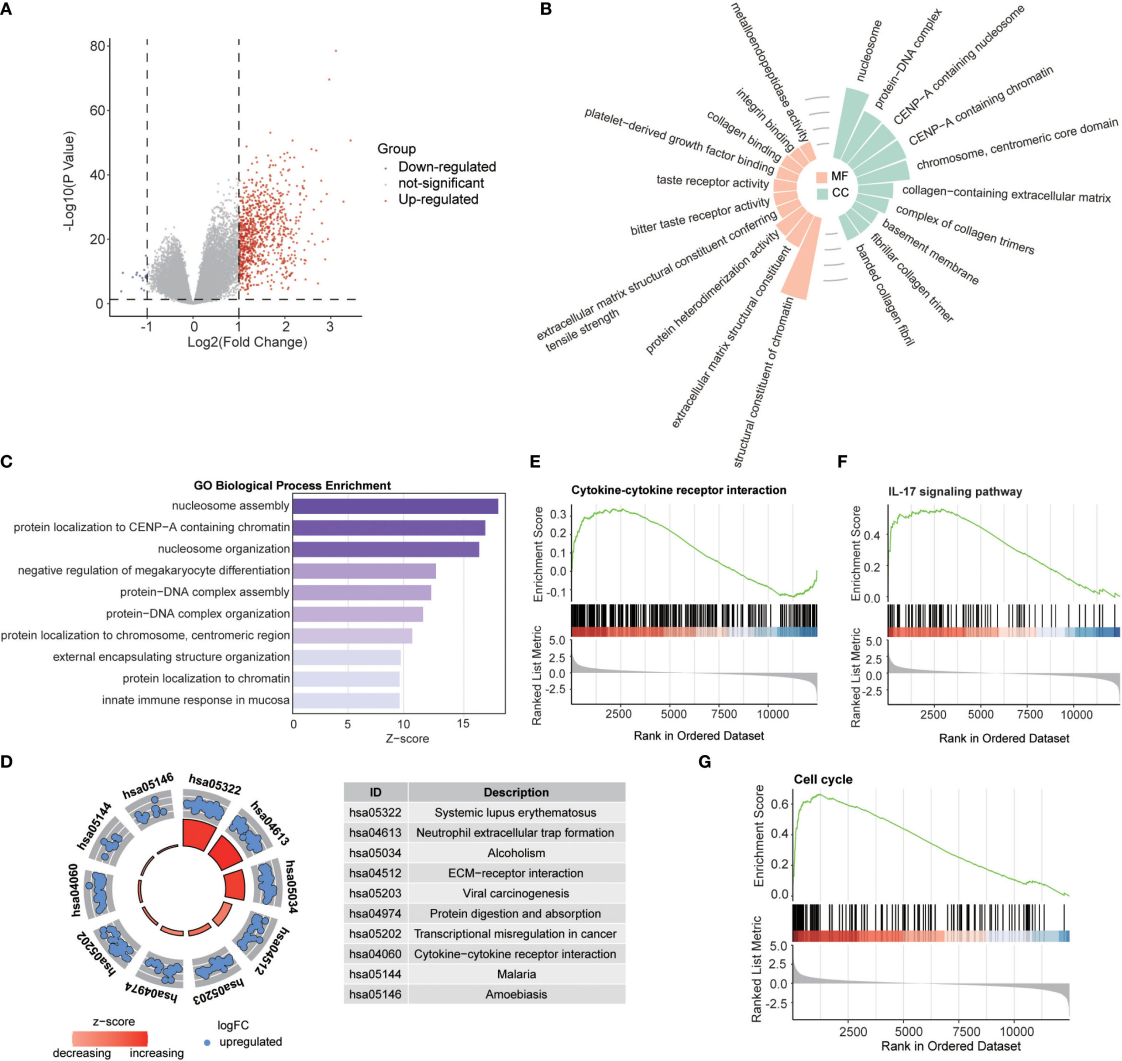
Biological functions of RBRGs. **(A)** Volcano plot showing differentially expressed genes between RBRGs-high and -low groups using the TCGA glioma cohort. **(B)** Radiographic histograms demonstrating RBRGs-related genes were subjected to GO enrichment analysis, including MF and CC. **(C)** Z-score plot demonstrating RBRGs-related genes were performed for GO enrichment analysis, including BP. **(D)** Chordal plot showing KEGG analysis of RBRGs-related genes. **(E–G)** GSEA analysis of RBRGs-related genes.

Building on the established role of RBRGs signature in glioma pathogenesis, we systematically interrogated their functional engagement with immune pathways through transcriptomic profiling of RBRGs-high versus -low groups. Differential expression analysis identified 888 significantly dysregulated genes (871 upregulated, 17 downregulated; threshold: |log_2_ FC| > 1, *P.adj* < 0.05; **Figure 5A**), with Gene Ontology (GO) analysis revealing compartmental enrichment in nucleosome, protein-DNA complex, and CENP-A containing nucleosome (**Figure 5B**). Molecular functions were dominated by structural constituent of chromatin, protein heterodimerization activity, and platelet-derived growth factor binding (**Figure 5B**). Biologically, these genes orchestrated immune activation through positive regulation of megakaryocyte differentiation, mucosal immune response, and innate immune response in mucosa (**Figure 5C**). KEGG pathway analysis confirmed enrichment in oncogenic-immune cascades, with systemic lupus erythematosus and neutrophil extracellular trap formation emerging as key nodes (**Figure 5D**). Gene set enrichment analysis (GSEA) further validated robust associations with cytokine-cytokine receptor interaction, IL-17 signaling pathway, and cell cycle, establishing a molecular paradigm wherein ribosome biogenesis-related genes modulate glioma progression through reciprocal immunoregulation (**Figures 5E–G**).

### The RBRGs signature shapes an immunosuppressive glioma microenvironment

**Figure 6:**
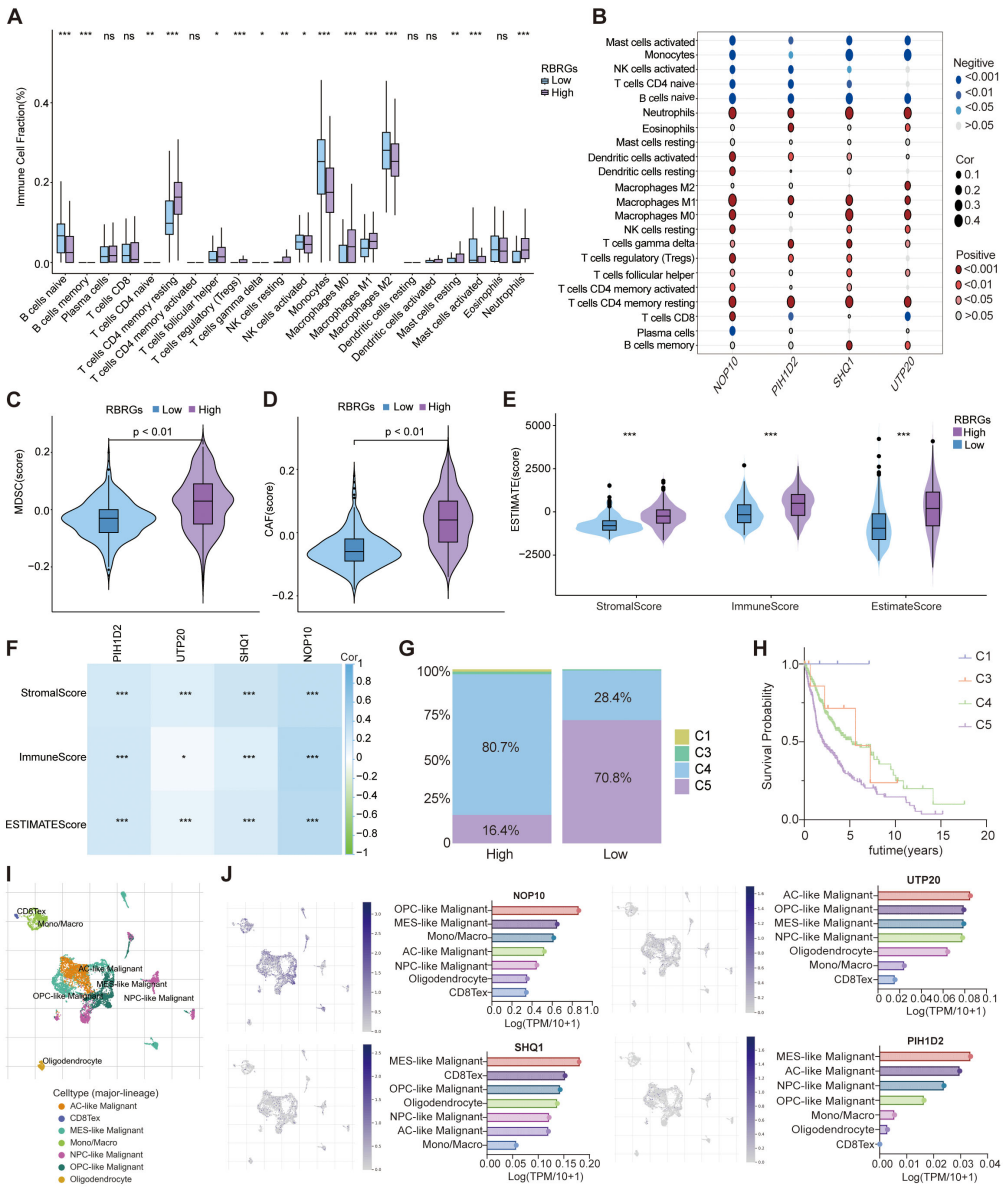
Role of RBRGs in the tumor microenvironment. **(A)** Differences in infiltration of immune cells between RBRGs-high and -low groups based on the CIBERSORTx algorithm. **(B)** Heatmap showing the correlation of NOP10, UTP20, SHQ1, and PIH1D2 with immune cells. **(C–E)** Differences in MDSC, CAF, and ESTIMATE scores between RBRGs-high and -low groups. **(F)** Heatmap showing the correlation of NOP10, UTP20, SHQ1, and PIH1D2 with ESTIMATE score. **(G)** Differences in immune subtypes between RBRGs-high and -low groups. **(H)** Kaplan-Meier curve showing the effect of immune subtype on overall survival of glioma patients. C1: wound healing, C3: inflammatory, C4: lymphocyte depleted, C5: immunologically quiet. **(I, J)** Single-cell analysis demonstrating the expression levels of NOP10, UTP20, SHQ1, and PIH1D2 in different cells based on the GSE131928 cohort. *p < 0.05, **p < 0.01, ***p < 0.001.

Capitalizing on the established link between RBRGs signature and immune pathways, we deployed the CIBERSORTx deconvolution algorithm to dissect immune cells distribution disparities across risk strata. Patients with high RBRGs score exhibited significant enrichment of immunosuppressive populations including M0/M1 macrophages, neutrophils, regulatory T cells (Tregs), γδ T cells, and resting memory CD4^+^ T cells, whereas those with low scores showed dominance of monocytes, activated NK cells, and mast cells (**Figure 6A**). Critically, the four core RBRGs NOP10, UTP20, SHQ1, and PIH1D2, demonstrated positive correlations with M0/M1 macrophages, neutrophils, Tregs, and γδ T cells, but inverse associations with monocytes and CD4^+^ naïve T cells (**Figure 6B**). TIDE algorithm analysis further confirmed elevated myeloid-derived suppressor cells (MDSCs) and cancer-associated fibroblasts (CAFs) enrichment in the RBRGs-high group (**Figures 6C, D**). ESTIMATE quantification revealed significantly increased Stromal, Immune, and ESTIMATE scores in the RBRGs-high group, with strong positive correlations between these scores and four core RBRGs expression (**Figures 6E, F**). Immunophenotypic stratification aligned with clinical outcomes: patients with low RBRGs score predominantly exhibited immune-favorable C5 subtype versus pro-tumorigenic C4 subtype in the RBRGs-high group (**Figures 6G, H**). Single-cell RNA sequencing localized PIH1D2, UTP20, and SHQ1 predominantly to malignant cells, while NOP10 co-expressed in monocytes/macrophages and exhausted T cells, suggesting direct tumor-immune crosstalk (**Figures 6I, J**). This multi-platform analysis establishes the RBRGs signature as a master regulator of immunosuppressive niche formation in glioma.

### The RBRGs signature precisely predicts immunotherapy efficacy

**Figure 7:**
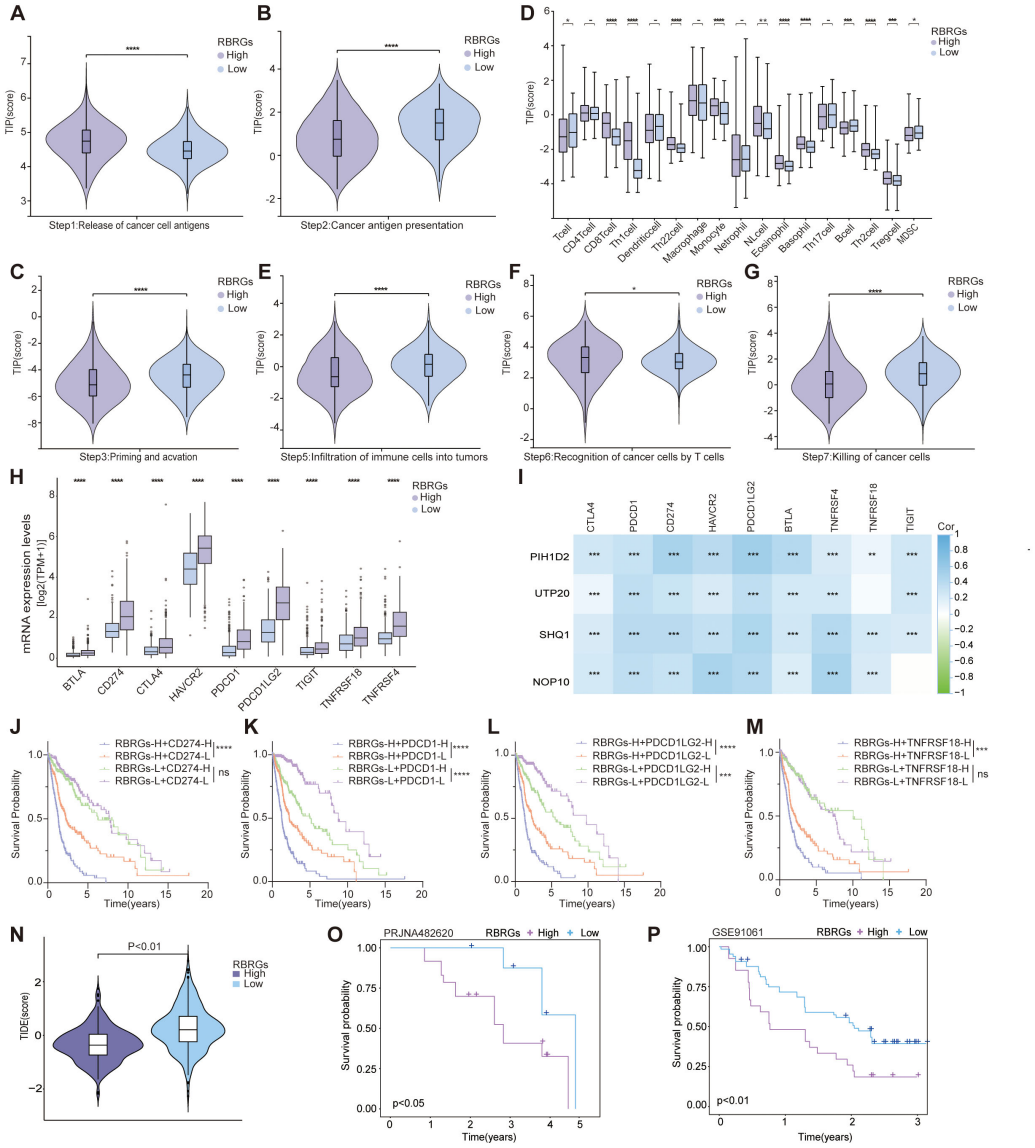
RBRGs predicted the efficacy of immunotherapy. Differences in cancer-immunity cycle between patients in the RBRGs-high and -low groups were based on the TCGA glioma cohort. **(A)** Step 1: release of cancer cell antigens. **(B)** Step 2: cancer antigen presentation. **(C)** Step 3: priming and activation. **(D)** Step 4: trafficking of immune cells to tumors. **(E)** Step 5: infiltration of immune cells into tumors. **(F)** Step 6: recognition of cancer cells by T cells. **(G)** Step7: killing of cancer cells. **(H)** Differential expression of immunosuppressive checkpoints in RBRGs-high and -low groups. **(I)** Heatmap demonstrating the correlation of NOP10, UTP20, SHQ1, and PIH1D2 with multiple immunosuppressive checkpoints. **(J-M)** Kaplan-Meier curves demonstrating RBRGs combined with CD274, PDCD1, PDCD1LG2, or TNFRSF18 respectively, to predict overall survival in glioma patients. **(N)** Differences in TIDE scores between RBRGs-high and -low groups. **(O, P)** Differences in survival between patients in the RBRGs-high and -low groups receiving immunotherapy were analyzed based on the glioma PRJNA482620 and melanoma GSE91061 cohorts. *p < 0.05, **p < 0.01, ***p < 0.001, ****p < 0.0001.

To investigate the regulatory role of RBRGs within the tumor immune microenvironment, we systematically compared cancer immunity cycle dynamics between RBRGs-high and -low groups in glioma. Results demonstrated that although glioma patients with high RBRGs score exhibited increased cancer antigen release, their antigen presentation and processing capacity was significantly impaired (**Figures 7A, B**). Concurrently, while high-RBRGs tumors recruited more immune cells to the peritumoral region, they displayed substantially reduced infiltration efficiency into the tumor parenchyma (**Figures 7C–E**). Notably, despite comparable T cell recognition capabilities toward cancer cells between the two groups, cytotoxic killing efficacy was markedly constrained in the RBRGs-high group (**Figures 7F, G**). Further investigation established a positive correlation between the RBRGs signature and immunosuppressive checkpoints, with multiple inhibitory genes significantly upregulated in the RBRGs-high group (**Figure 7H**). Specifically, NOP10, UTP20, SHQ1, and PIH1D2 showed strong positive correlations with these immunosuppressive markers (**Figure 7I**). Kaplan-Meier survival analyses confirmed significantly poorer prognosis in RBRGs high-risk patients overexpressing CD274, PDCD1, PDCD1LG2, and TNFRSF18 (**Figures 7J–M**). Ultimately, validation using the TIDE algorithm and glioma immunotherapy cohort PRJNA482620, along with cross-cancer immunotherapy data from the melanoma cohort GSE91061, demonstrated that patients with high RBRGs expression exhibited elevated TIDE scores, shorter survival durations, and reduced immunotherapy response rates. These findings collectively support the RBRGs signature as a potential predictor of immunotherapy resistance (**Figures 7N–P**).

### Integrative analysis of the RBRGs signature and genomic alterations

**Figure 8:**
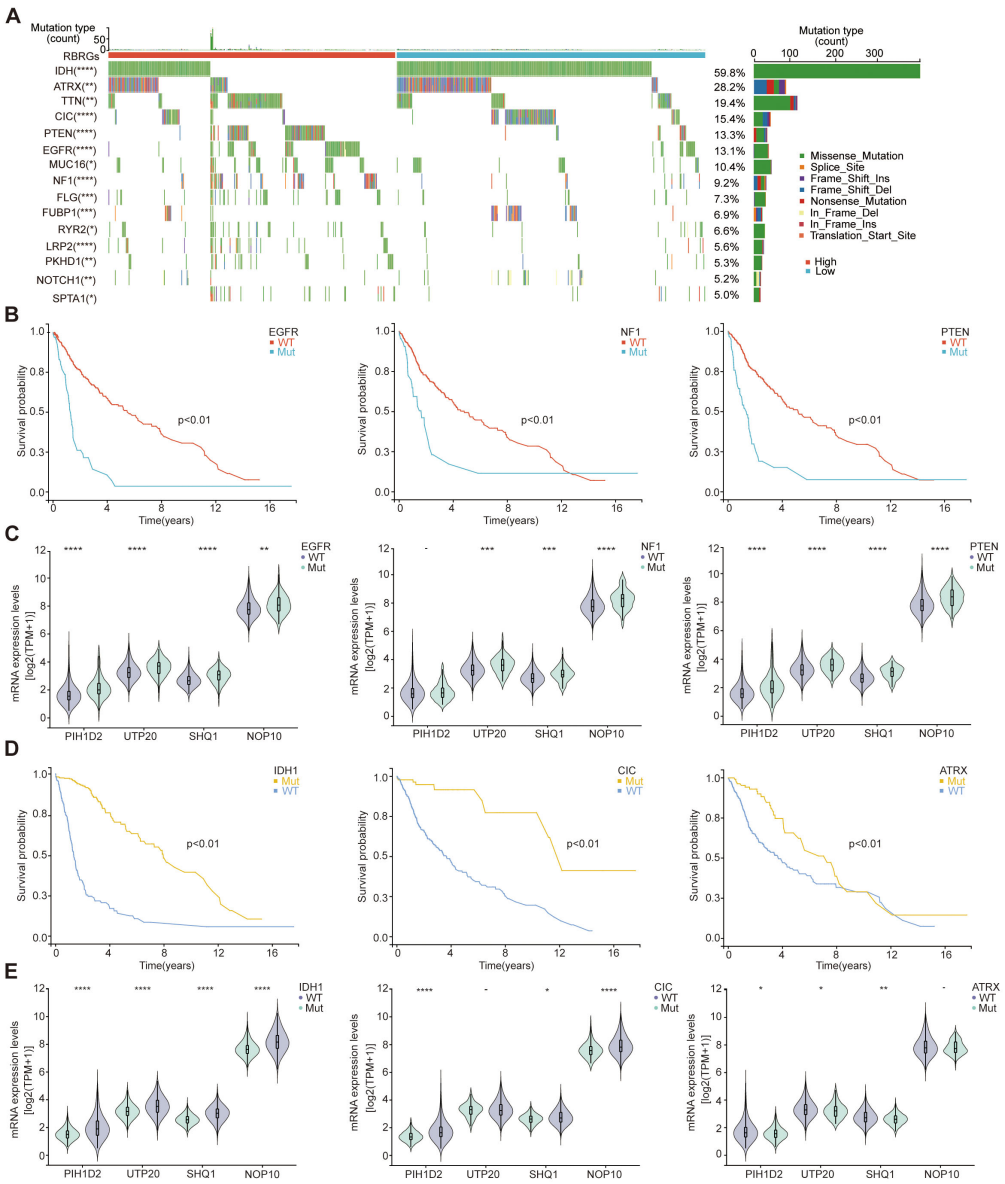
Genetic mutations. **(A)** The oncoplot depicted genetic mutations differences in glioma patients between RBRGs-high and -low groups. **(B)** Kaplan-Meier curves demonstratingthe difference in overall survival of glioma patients between the EGFR, NF1 and PTEN mutation and wild-type groups. **(C)** Differential expression of NOP10, UTP20, SHQ1, and PIH1D2 between EGFR, NF1and PTEN mutant and wild-type groups, respectively. **(D)** Kaplan-Meier curves demonstrating the difference in overall survival of glioma patients between the IDH1, CIC and ATRX mutation and wild-type groups. **(E)** Differential expression of NOP10, UTP20, SHQ1, and PIH1D2 between IDH1, CIC and ATRX mutant and wild-type groups, respectively.*p < 0.05, **p < 0.01, ***p < 0.001, ****p < 0.0001.

Given the established association between genetic alterations and gliomagenesis, we further investigated the role of RBRGs in genetic mutations and glioma progression through comprehensive mutational profiling. Waterfall plot analysis revealed that mutations in key driver genes (EGFR, PTEN, and NF1) correlated with shorter overall survival in glioma patients, concurrent with elevated expression of core RBRGs components (NOP10, UTP20, SHQ1, and PIH1D2) within these mutational subgroups, with the notable exception of sustained PIH1D2 expression in the NF1-mutant subgroup (**Figures 8A–C**). Conversely, mutations in IDH1, CIC, and ATRX emerged as favorable prognostic markers, demonstrating significantly reduced expression of these RBRGs elements, with the notable exception of sustained UTP20 expression in the CIC-mutant subgroup and sustained NOP10 expression in the ATRX-mutant subgroup (**Figures 8D, E**).

### Role of the RBRGs signature in tumor stemness, genomic heterogeneity, and drug sensitivity prediction

**Figure 9:**
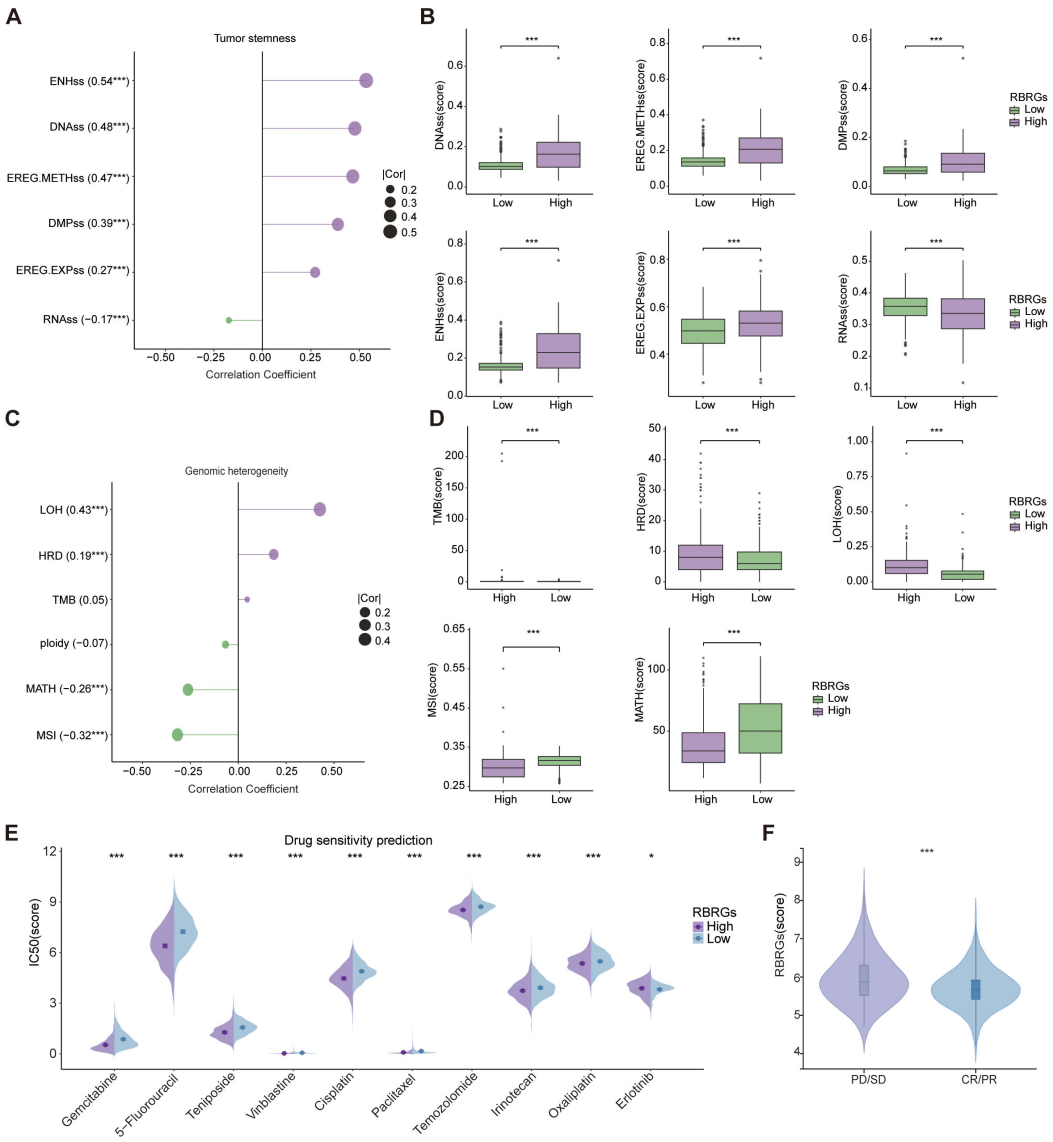
Tumor stemness, genomic heterogeneity, and drug sensitivity analyses. **(A)** Correlation of RBRGs with stemness-related metrics (DNAss, EREG-METHss, DMPss, ENHss, RNAss, EREG-EXPss). **(B)** Differences in stemness metrics between RBRGs-high and -low groups. **(C)** Correlation of RBRGs with genomic heterogeneity metrics (TMB, MATH, MSI, Ploidy, HRD, LOH). **(D)** Differences in TMB, HRD, LOH, MSI, and MATH between groups. **(E)** Sensitivity differences to chemotherapeutic agents between RBRGs-high and -low groups. **(F)** Differences in RBRGs expression between PD/SD and PR/CR glioma patients treated with radiotherapy. *p < 0.05, ***p < 0.001.

Our investigation delineates the multifaceted role of the RBRGs signature in modulating oncogenic stemness, genomic heterogeneity, and therapeutic vulnerability. The RBRGs signature demonstrated significant positive correlations with established stemness indices, including DNA stemness score (DNAss), epiregulin methylation score (EREG-METHss), differentially methylated probes score (DMPss), enhancer score (ENHss), and epiregulin expression score (EREG-EXPss), but negatively with RNAss (**Figures 9A, B**). Further analysis revealed divergent associations between the RBRGs signature and genomic heterogeneity metrics: while positively correlated with tumor mutation burden (TMB), homologous recombination deficiency (HRD), and loss of heterozygosity (LOH), it exhibited inverse relationships with mutant-allele tumor heterogeneity (MATH) and microsatellite instability (MSI), findings consistently validated in the RBRGs-high group (**Figures 9C, D**). Drug sensitivity profiling demonstrated that patients with elevated RBRGs score exhibited higher half-maximal inhibitory concentrations (IC50) for erlotinib, yet lower IC50 values for gemcitabine, 5-fluorouracil, teniposide, vinblastine, cisplatin, paclitaxel, temozolomide, irinotecan, and oxaliplatin (**Figure 9E**). Moreover, glioma patients exhibiting disease progression or stable disease (PD/SD) following radiotherapy presented significantly elevated RBRGs score compared to those achieving partial or complete response (PR/CR), underscoring its utility in predicting treatment refractoriness (**Figure 9F**).

### UTP20 as a key driver of glioma tumorigenesis

**Figure 10:**
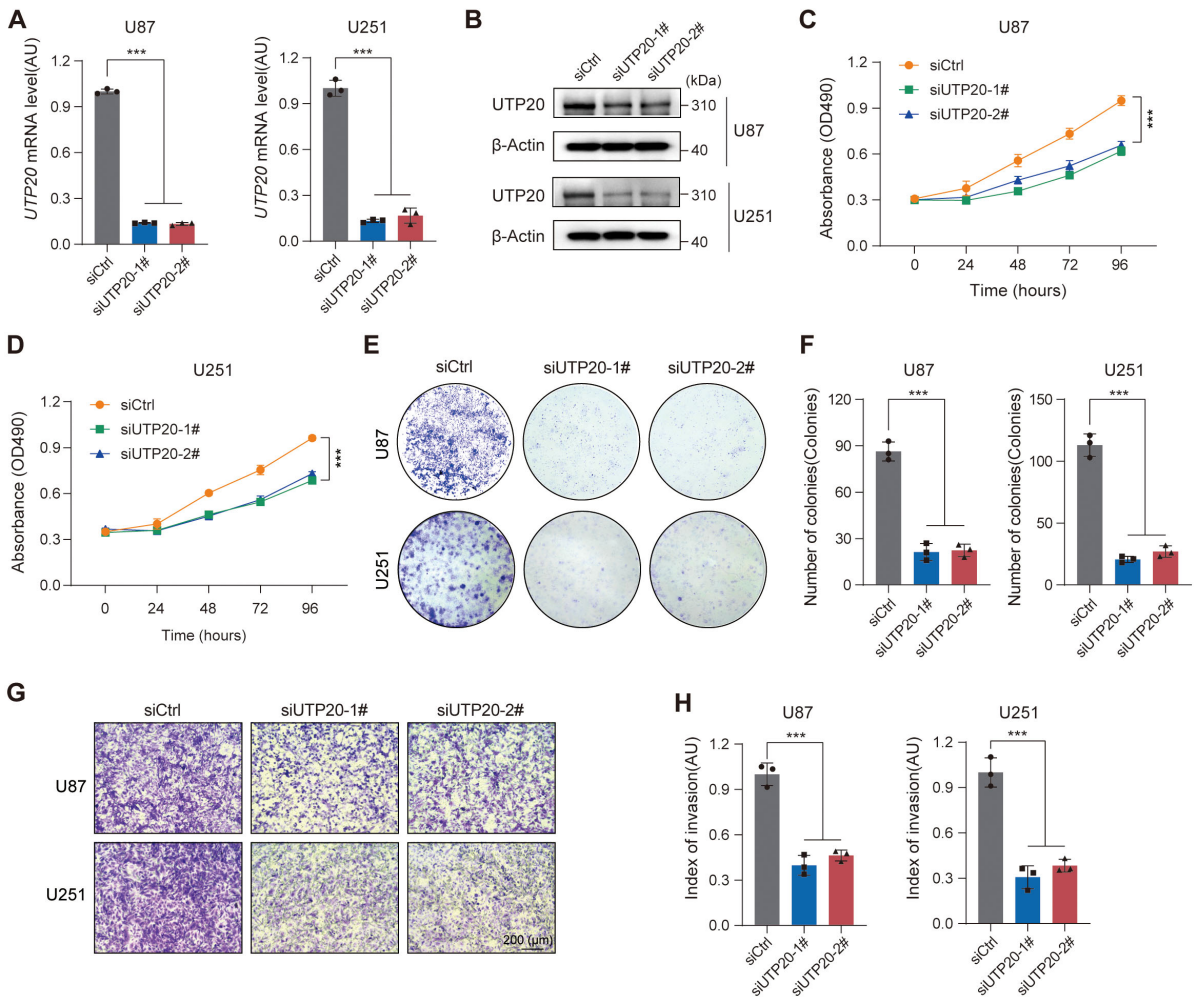
Knockdown of UTP20 expression inhibited proliferation and invasion of glioma U251 and U87 cells *in vitro*. **(A)** UTP20 mRNA levels in U87 and U251 cells after knockdown. **(B)** Western Blot showing UTP20 protein levels in U87 and U251 cells after knockdown. **(C, D)** MTS assay for cell proliferation in U87 and U251 after UTP20 knockdown. **(E)** Colony formation assay showing the number of colonies in U87 and U251 after UTP20 knockdown. **(F)** Quantification of colonies formed in U87 and U251 cells after UTP20 knockdown. **(G)** Transwell invasion assay showing cell migration in U87 and U251 cells. **(H)** Quantification of cell migration in U87 and U251 cells. ***p < 0.001. One-way ANOVA with Tukey’s test for **(A, F)**, and **(H)** Two-way ANOVA with Tukey’s test for **(C, D)**.

Given the potential clinical significance of the RBRGs signature in glioma, we focused on UTP20, the gene with the highest hazard ratio in the multivariate Cox regression model and whose functional role in glioma remains poorly understood. To investigate its role in glioma progression, we established UTP20-knockdown models in glioma cell lines. qPCR and Western blot analyses confirmed effective knockdown of UTP20 expression in both U87 and U251 glioma cells (**Figures 10A, B**). Functional assays revealed that UTP20 knockdown markedly inhibited glioma cell proliferation, as demonstrated by MTS assays and colony formation experiments (**Figures 10C–F**). Moreover, transwell invasion assays showed that silencing UTP20 significantly reduced the invasive capacity of glioma cells (**Figures 10G–H**). Collectively, these findings suggest that UTP20, as a component of the ribosome biogenesis-related gene signature, plays a critical oncogenic role in glioma progression.

## Discussion

Glioma is a common primary malignancy of the central nervous system, with peak incidence observed in middle-aged to elderly individuals (45–70 years) (17), and continues to be associated with a dismal prognosis (18). This unfavorable outcome stems from the tumor’s intrinsic biological aggressiveness, marked by diffuse infiltration into neural parenchyma, as well as significant therapeutic challenges, including the restrictive nature of the blood-brain barrier, pronounced intra- and intertumoral heterogeneity, and the frequent development of resistance to standard treatment regimens. Collectively, these factors underscore the limitations of current therapeutic approaches. As such, the identification and validation of novel molecular biomarkers is not merely a tool for prognostic stratification, but a foundational step for advancing precision oncology and fostering the development of effective, mechanism-based therapies against this treatment-resistant malignancy.

Ribosome biogenesis has emerged as a fundamental post-transcriptional regulatory axis in glioma pathobiology, orchestrating malignant progression through metabolic reprogramming, proliferative signaling, and maintenance of cellular stemness. Coordinated dysregulation of RB components actively drives oncogenic transformation; for example, NSUN5 upregulation accelerated protein synthesis in glioblastoma (GBM) (19), whereas WDR12 silencing disrupted ribosome biogenesis and inhibits proliferation in glioma stem cells (GSCs) (20). As a critical ribosomal protein, RPS14 promoted tumorigenesis through activation of p53-dependent signaling pathways (21), MRPS23 promoted neoplastic survival and motility (22), and RPL34 knockdown implicated JAK/STAT3 signaling as essential for glioma cell proliferation and migration (23). Notably, RB also sustained the tumor-supportive microenvironment by enabling cellular reprogramming; for instance, ribosomal incorporation and RPS6 overexpression induced stem-like phenotypic transitions in GBM cells, enhancing resistance to therapy (24). Collectively, these findings position ribosome biogenesis not merely as a metabolic facilitator, but as a central regulatory hub that integrates the defining hallmarks of glioma. Dissecting its control mechanisms may uncover fundamental drivers of gliomagenesis and reveal actionable therapeutic targets.

This integrative analysis of TCGA-derived clinical and molecular profiles established ribosome biogenesis-related genes as pivotal determinants of glioma prognosis, with the four-gene signature (NOP10, UTP20, SHQ1, and PIH1D2) demonstrating significant overexpression in tumor tissues and robust survival stratification capacity. Beyond validating ribosomal metabolism as a critical driver in glioma pathogenesis, the model mechanistically connects tumor metabolic reprogramming with clinical outcomes, offering a clinically actionable framework for risk stratification and personalized therapeutic decision-making. Notably, this approach surpasses traditional prognostic models by incorporating multidimensional features of tumor microenvironment dynamics, immunotherapy responsiveness, genomic heterogeneity landscapes, stemness indices, and pharmacological vulnerability profiles (25), thereby achieving enhanced predictive accuracy and biological relevance. This comprehensive investigation of ribosome biogenesis within the extended spectrum of tumorigenic mechanisms not only uncovers novel molecular drivers of gliomagenesis but establishes a precision oncology framework focused on targeting RB-mediated dependencies in diverse glioma subtypes.

A pivotal innovation of this study resides in the development of a clinically integrated nomogram that combines the RBRG-based risk signature with key clinicopathological variables, including age, WHO grade, IDH mutational status, and 1p/19q codeletion, yielding significantly enhanced prognostic discrimination (C-index = 0.841). The model exhibited strong temporal predictive performance, with time-dependent ROC analyses yielding AUC values consistently above 0.700 across 1- to 5-year survival intervals. Critically, decision curve analysis (DCA) substantiated that incorporating the RBRG signature into the nomogram significantly improves net clinical benefit over conventional prognostic tools for 3- and 5-year survival prediction, thereby establishing it as a quantitatively validated and clinically actionable instrument for individualized therapeutic decision-making in neuro-oncology practice.

The profoundly immunosuppressive glioma microenvironment, characterized by regulatory T cell-mediated impairment of cytotoxic lymphocyte function (26), is critically modulated by ribosome biogenesis pathways. Our findings establish the RBRGs signature as central orchestrators of this immunosuppressive microenvironment, with elevated RBRGs scores significantly correlating with increased infiltration of tumor-promoting macrophages, myeloid-derived suppressor cells (MDSCs), and cancer-associated fibroblasts (CAFs). Mechanistically, overexpression of RPS3A drives E2F1-dependent transcriptional activation of CSF1, which recruits tumor-associated macrophages and induces M2 polarization via autophagy-mediated reprogramming, a process that directly facilitates glioma progression (27). In contrast, the conserved RNA-binding protein SRBD1 exhibits tumor-suppressive effects through ectopic expression, which reduces M2 TAM density and inhibits tumor growth (28), highlighting the dual regulatory capacity of ribosomal machinery.Importantly, M2-polarized cells sustain immunosuppression by secreting IL-10, TGF-β, and VEGF (29), creating a self-amplifying circuit wherein ribosome biogenesis-related genes serve as master regulators that maintain the pro-tumoral microenvironment through coordinated immune cell recruitment, phenotypic polarization, and cytokine dysregulation, thereby revealing novel actionable therapeutic targets for immune-based treatments.

The evolving recognition of the central nervous system’s lymphatic architecture has intensified focus on immunotherapeutic strategies for therapy-resistant glioma. Notably, interventions such as Veledimex-regulated IL-12 gene therapy have demonstrated clinical promise by promoting CD8^+^ T cell recruitment in recurrent GBM (30), while combined PD-1/VEGF inhibition (31) and the FDA-approved anti-VEGF monoclonal antibody bevacizumab (32) validated multimodal targeting strategies. Paradoxically, our findings revealed that ribosome biogenesis fundamentally constrains these therapeutic advances, as elevated RBRGs score correlated significantly with diminished immunotherapy response rates and attenuated overall clinical benefit, indicating direct involvement in immune evasion pathways. This immunosuppressive hub functions via mitochondrial rRNA regulatory elements that orchestrate tumor microenvironment complexity and prognostic determinants (33), while nucleolar protein 14 (NOP14) concurrently modulated CD8^+^ T cell infiltration and epithelial-mesenchymal transition (EMT) networks (34). Critically, this mechanistic convergence also positions ribosome biogenesis-related genes as master regulators of therapeutic resistance. They intrinsically limit the efficacy of emerging modalities including immune checkpoint blockade (ICB) (35) and anti-angiogenic agents, while also undermining promising gene therapies currently under clinical investigation (36). Consequently, targeting ribosome biogenesis emerges not merely as a complementary strategy but as an essential precondition for overcoming the immunosuppressive barriers that currently constrain neuro-oncological immunotherapy.

A key advancement of this study was the functional characterization of UTP20 as a clinically relevant oncogenic driver in glioma. We revealed that UTP20 facilitated tumor progression by promoting cellular proliferation and invasion, thereby extending the known oncogenic activity of its homolog 1A6/DRIM, previously shown to enhance cell growth via RNA polymerase I-mediated transcriptional activation (37). Previous investigations have established that DRIM, functioning as both a nuclear protein and cytoskeleton-associated factor, promotes angiogenesis by activating endothelial cell phosphorylation pathways, specifically affecting downstream effectors p-FAK and p-STAT3 (38). Our work provided the first mechanistic evidence demonstrating that UTP20 knockdown in glioma cell models directly inhibited tumor growth and proliferation capacity. This finding not only underscored UTP20’s fundamental role in oncogenic programs but also established its tissue-specific functional significance in central nervous system malignancies. Crucially, our experimental validation substantiates the computational prognostic framework while mechanistically positioning UTP20 as a therapeutically actionable vulnerability, thereby revealing its druggable potential through direct demonstration of tumorigenic dependency in glioma pathogenesis.

This work established a first-in-class integrative prognostic model for glioma that harnesses machine learning algorithms to synthesize genomic signatures, tumor microenvironment dynamics, and experimental validation into a cohesive predictive framework, delivering exceptional accuracy in patient risk stratification. Beyond its computational advancement, the model offers mechanistic insight into previously elusive aspects of glioma biology, particularly ribosome biogenesis-driven metabolic reprogramming and immune evasion, and establishes a clinically translatable platform for precision neuro-oncology. Future multi-center validation efforts will be critical to advancing its clinical adoption, facilitating the translation of molecular discoveries into personalized therapeutic strategies.

## Limitations

Although this study has yielded promising preliminary results, several limitations remain. First, the model was developed based on retrospective cohort data, and its clinical applicability requires further validation through prospective clinical studies. Second, additional key ribosome biogenesis-related genes (RBRGs) should be incorporated to improve the robustness and generalizability of the model. Moreover, the biological functions of the identified genes have not been fully elucidated, particularly *in vivo*. To address this gap, we plan to establish a U87 stable cell line with UTP20 knockdown and utilize an orthotopic intracranial xenograft model in nude mice to investigate the *in vivo* impact of UTP20 on glioma cells. We will further examine whether UTP20 regulates glioma function by modulating the translation of specific mRNAs through a series of experimental approaches, including nascent protein synthesis assays, polysome profiling, ribosome component isolation, ribosome sequencing, and UTP20 RIP-seq. Notably, the mechanisms by which ribosomal biogenesis influences the tumor immune microenvironment, especially immune cell infiltration, remain unclear, warranting further mechanistic investigations.

## Conclusion

In this study, a glioma prognostic model incorporating ribosome biogenesis-related genes was constructed and validated. By systematically integrating clinical parameters, immune microenvironment features, genomic heterogeneity, and drug sensitivity, the model demonstrated strong predictive performance. It offers an effective tool for risk stratification in glioma patients and provides a theoretical foundation for developing individualized treatment strategies. Experimental validation further confirmed the pivotal role of UTP20 in glioma progression, underscoring its potential as a novel therapeutic target and biomarker. Overall, this study emphasizes the value of integrating molecular signatures with machine learning approaches to enhance prognostic accuracy and inform clinical decision-making in glioma.

## Data Availability

The original contributions presented in the study are included in the article/**Supplementary Material**. Further inquiries can be directed to the corresponding authors.
