# Supplementary material for: Ribosome biogenesis-related gene signature predicts prognosis and immune landscape in glioma and identifies UTP20 as a therapeutic target
Source: Front Immunol. 2025 Oct 8;16:1680667. doi: 10.3389/fimmu.2025.1680667 (PMC12540490; doi:10.3389/fimmu.2025.1680667)
Supplement: Supplementary file 2 [file Table2.docx]

The Sequences of the siRNAs were as follows:

siUTP20-1#

Sense: 5’-CCUCGAUCCUGGAGACUCATT-3’

Antisense: 5’-UGAGUCUCCAGGAUCGAGGTT-3’

siUTP20-2#

Sense: 5’-CCUCGUUAUCAUAUCUUUATT-3’

Antisense: 5’-UAAAGAUAUGAUAACGAGGTT-3’

The following primers were used for qPCR:

GAPDH-F: 5’-GTCTCCTCTGACTTCAACAGCG-3’,

GAPDH-R: 5’-ACCACCCTGTTGCTGTAGCCAA-3’;

UTP20-F: 5’- GAGACTTTCCAGACCATCACCTC-3’,

UTP20-R: 5’- ACTCATCAGGCACATGCTGGCA-3’.
